# Liquid argon light collection and veto modeling in GERDA Phase II

**DOI:** 10.1140/epjc/s10052-023-11354-9

**Published:** 2023-04-24

**Authors:** M. Agostini, A. Alexander, G. R. Araujo, A. M. Bakalyarov, M. Balata, I. Barabanov, L. Baudis, C. Bauer, S. Belogurov, A. Bettini, L. Bezrukov, V. Biancacci, E. Bossio, V. Bothe, R. Brugnera, A. Caldwell, S. Calgaro, C. Cattadori, A. Chernogorov, P. -J. Chiu, T. Comellato, V. D’Andrea, E. V. Demidova, A. Di Giacinto, N. Di Marco, E. Doroshkevich, F. Fischer, M. Fomina, A. Gangapshev, A. Garfagnini, C. Gooch, P. Grabmayr, V. Gurentsov, K. Gusev, J. Hakenmüller, S. Hemmer, W. Hofmann, M. Hult, L. V. Inzhechik, J. Janicskó Csáthy, J. Jochum, M. Junker, V. Kazalov, Y. Kermaïdic, H. Khushbakht, T. Kihm, K. Kilgus, I. V. Kirpichnikov, A. Klimenko, K. T. Knöpfle, O. Kochetov, V. N. Kornoukhov, P. Krause, V. V. Kuzminov, M. Laubenstein, B. Lehnert, M. Lindner, I. Lippi, A. Lubashevskiy, B. Lubsandorzhiev, G. Lutter, C. Macolino, B. Majorovits, W. Maneschg, L. Manzanillas, G. Marshall, M. Miloradovic, R. Mingazheva, M. Misiaszek, M. Morella, Y. Müller, I. Nemchenok, M. Neuberger, L. Pandola, K. Pelczar, L. Pertoldi, P. Piseri, A. Pullia, L. Rauscher, M. Redchuk, S. Riboldi, N. Rumyantseva, C. Sada, S. Sailer, F. Salamida, S. Schönert, J. Schreiner, M. Schütt, A. -K. Schütz, O. Schulz, M. Schwarz, B. Schwingenheuer, O. Selivanenko, E. Shevchik, M. Shirchenko, L. Shtembari, H. Simgen, A. Smolnikov, D. Stukov, S. Sullivan, A. A. Vasenko, A. Veresnikova, C. Vignoli, K. von Sturm, A. Wegmann, T. Wester, C. Wiesinger, M. Wojcik, E. Yanovich, B. Zatschler, I. Zhitnikov, S. V. Zhukov, D. Zinatulina, A. Zschocke, A. J. Zsigmond, K. Zuber, G. Zuzel, Gerda collaboration

**Affiliations:** 1 INFN Laboratori Nazionali del Gran Sasso, Assergi, Italy; 2 INFN Laboratori Nazionali del Gran Sasso and Gran Sasso Science Institute, Assergi, Italy; 3 INFN Laboratori Nazionali del Gran Sasso and Università degli Studi dell’Aquila, L’Aquila, Italy; 4 INFN Laboratori Nazionali del Sud, Catania, Italy; 5 Institute of Physics, Jagiellonian University, Cracow, Poland; 6 Institut für Kern- und Teilchenphysik, Technische Universität Dresden, Dresden, Germany; 7 Joint Institute for Nuclear Research, Dubna, Russia; 8 European Commission, JRC-Geel, Geel, Belgium; 9 Max-Planck-Institut für Kernphysik, Heidelberg, Germany; 10 Department of Physics and Astronomy, University College London, London, UK; 11 INFN Milano Bicocca, Milan, Italy; 12 Dipartimento di Fisica, Università degli Studi di Milano and INFN Milano, Milan, Italy; 13 Institute for Nuclear Research of the Russian Academy of Sciences, Moscow, Russia; 14 Institute for Theoretical and Experimental Physics, NRC “Kurchatov Institute”, Moscow, Russia; 15 National Research Centre “Kurchatov Institute”, Moscow, Russia; 16 Max-Planck-Institut für Physik, Munich, Germany; 17 Physik Department, Technische Universität München, Munich, Germany; 18 Dipartimento di Fisica e Astronomia, Università degli Studi di Padova, Padua, Italy; 19 INFN Padova, Padua, Italy; 20 Physikalisches Institut, Eberhard Karls Universität Tübingen, Tübingen, Germany; 21 Physik-Institut, Universität Zürich, Zurich, Switzerland; 22 Present Address: Duke University, Durham, NC USA; 23 Present Address: Leibniz-Institut für Kristallzüchtung, Berlin, Germany; 24 Present Address: Nuclear Science Division, Berkeley, USA; 25 Present Address: Université Paris-Saclay, CNRS/IN2P3, IJCLab, 91405 Orsay, France; 26 NRNU MEPhI, Moscow, Russia; 27 Moscow Inst. of Physics and Technology, Dolgoprudny, Russia; 28 Dubna State University, Dubna, Russia

## Abstract

The ability to detect liquid argon scintillation light from within a densely packed high-purity germanium detector array allowed the Gerda experiment to reach an exceptionally low background rate in the search for neutrinoless double beta decay of ${}^{76}$Ge. Proper modeling of the light propagation throughout the experimental setup, from any origin in the liquid argon volume to its eventual detection by the novel light read-out system, provides insight into the rejection capability and is a necessary ingredient to obtain robust background predictions. In this paper, we present a model of the Gerda liquid argon veto, as obtained by Monte Carlo simulations and constrained by calibration data, and highlight its application for background decomposition.

## Introduction

Provided with an array of germanium detectors, made from isotopically enriched high-purity germanium (HPGe) material suspended in a clean liquid argon (LAr) bath, the Germanium Detector Array (Gerda) experiment set out to probe the neutrino’s particle nature in a search for the neutrinoless double beta (${0{\nu \beta \beta }}$) decay of ${}^{76}$Ge [1]. The ability to detect scintillation light emerging from coincident energy depositions in the LAr, combined with pulse shape discrimination (PSD) techniques [2], allowed to cut the background level of the second phase (Phase II ) to a record low [3]. No signal was found, which translates into one of the most stringent lower limits on the half-life of the ${0{\nu \beta \beta }}$decay of ${}^{76}$Ge at $1.8 \cdot 10^{26}$ years at 90% C.L. [4].

Based on dedicated Monte Carlo simulations of the scintillation light propagation, the model of the LAr veto rejection grants insight into the light collection from various regions of the highly heterogeneous setup. The full methodology and its first application are described in this document, which is structured as follows: Sect. 2 offers a brief description of the Gerda instrumentation, focusing on the Phase II light read-out system. In Sect. 3 the connection between photon detection probabilities and event rejection is made. Section 4 summarizes the Monte Carlo implementation and the chosen optical properties, while Sect. 5 describes the tuning of the model parameters on calibration data. In Sect. 6 photon detection probability maps are introduced, and in Sect. 7 their application for background decomposition is highlighted. In Sect. 8 conclusions are drawn.

## Instrumentation

The Gerda experimental site was the Hall A of the INFN Laboratori Nazionali del Gran Sasso (LNGS) underground laboratory in central Italy. Equipped with a large-scale shielding infrastructure – a 64 $\hbox {m}^{3}$ cryostat inside a 590 $\hbox {m}^{3}$ water tank – Gerda enclosed a low-background 5.0-grade LAr environment, which from December 2015 to November 2019 gave home to the heart of Phase II : 40, later 41, HPGe detectors in a 7-string array configuration, surrounded by a light read-out instrumentation. The veto design comprised two sub-systems: low-activity photomultiplier tubes (PMTs) [5] and wavelength-shifting (WLS) fibers coupled to silicon photomultipliers (SiPMs) [6]. The latter was upgraded in spring 2018. The reader is referred to [7] for a detailed description of the Gerda experimental setup.

**Fig. 1:**
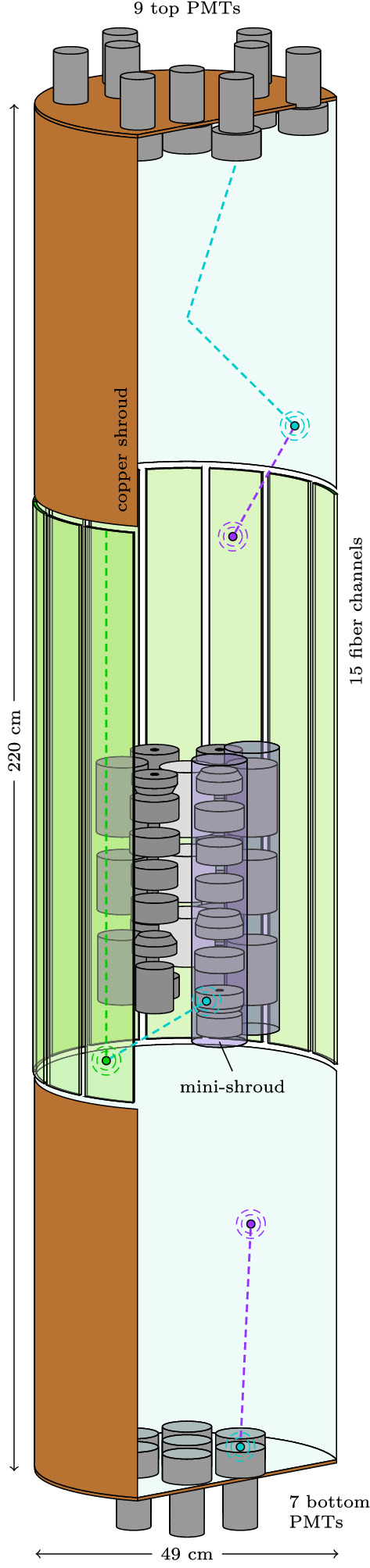
LAr veto instrumentation concept. Transport of light signals towards the PMTs or SiPMs relies on WLS processes in the TPB layers or optical fibers. Several potential light paths are indicated. Support structure details, electronics as well as individual fibers are not drawn

The 3$''$ Hamamatsu R11065-20 Mod PMTs were selected for their performance at LAr temperature and enhanced radiopurity (< 2 mBq activity in both ${}^{228}$Th and ${}^{226}$Ra) [7–9]. Still, they contributed significantly to the background within the inner Phase II setup and were thus placed at >1 m from any HPGe detector, giving – together with the limited cryostat entrance width – the LAr instrumentation its elongated cylindrical shape. With space for support and calibration sources to enter, 3 off-center groups of 3 PMTs each were installed on top, whereas the bottom plate held 7 centrally mounted PMTs. Each PMT’s entrance window was covered with tetraphenyl butadiene (TPB) embedded in polystyrene, in order to shift the incident vacuum-ultraviolet (VUV) scintillation light from LAr to a detectable wavelength. On the inside, the horizontal copper support plates were covered with a highly reflective TPB-painted VM2000 multi-layer polymer, whereas lateral guidance of light towards the top/bottom was ensured by a TPB dip-coated diffuse-reflecting  polytetrafluoroethylene (PTFE) foil [10], stitched to the 100 mm-thin copper shrouds. A sketch of the setup is shown in Fig. 1.

With typical SiPMs having a photo-sensitive area of $\mathcal {O}$(1) $\hbox {cm}^2$, large-scale installations of >1 $\hbox {m}^2$ photo coverage still represent a technological challenge [11]. Nonetheless, coupled to WLS fibers of < 0.1 mBq/kg activity in both ${}^{228}$Th and ${}^{226}$Ra, which serve as radio-pure light collectors [12], the detection power of a single device is largely enhanced. The active chip size of the KETEK PM33100 SiPMs is 3$\times$3 $\hbox {mm}^{2}$, they feature 100 $\upmu \,\hbox {m}$ micro cell pitch and were purchased “in die”, i.e. without packaging, allowing for a custom low-activity housing. Each of the 15 channels was comprised of 6 SiPMs, connected in parallel on copper-laminated PTFE holders and cast into optical cement, amounting to a total active surface of 8.1 $\hbox {cm}^{2}$. The doubly-cladded BCF-91A fibers of square-shaped 1$\times$1 $\hbox {mm}^{2}$ cross section, were covered with TPB by evaporation, routed vertically to cover the central veto section, bent by 180$^{\circ }$ at the bottom and coupled to different SiPMs on both top ends. Guidance of the individual fibers was ensured by micro-machined copper holders, attempting to keep them at a 45$^{\circ }$ rotation, facing their full $\sqrt{2}$ mm-diagonal towards the center. The total length of the 405 fibers was about 730 m.

Each of the 40 cm-long HPGe strings was enclosed in a nylon “mini shroud”, transparent to visible light, covered on both sides with TPB. It provided a mechanical barrier that limited the accumulation of ${}^{42}$K ions – a progeny of cosmogenic ${}^{42}$Ar – on the HPGe detector surfaces [13].

The data acquisition of the entire array, based on SIS3301 Struck [14] FADCs, including the LAr veto photo sensors, was triggered once the signal of a single HPGe detector exceeded a pre-set online threshold. No independent trigger on the light read-out was implemented. The veto condition was evaluated offline, allowing for time-dependent channel-specific thresholds right above the respective noise pedestals and an anti-coincidence window that takes into account the characteristic scintillation emission timing as well as the HPGe detector signal formation dynamics. The typical thresholds were set at about 0.5 photo-electrons within $-1$ to + 5 $\upmu \hbox {s}$ around the HPGe detector trigger. Any light signal over threshold in any channel was sufficient to classify the event as background.

## Photon detection probabilities

Upon interaction of ionizing radiation, ultra-pure LAr scintillates with a light yield of 40 photons/keV [15]. There is an ongoing discussion whether this number could be smaller [16], but in any case, the actual light output is strongly reduced in the presence of trace contaminants and a priori not precisely known for many experiments, including Gerda. An estimate based on the measured triplet lifetime of the argon excimer state of about 1.0 $\upmu \,\hbox {s}$ [7], limits the Gerda light yield to < 71% of the nominal pure-argon value, or < 28 photons/keV.^1^

Given a light yield $L'$ of this order, the number of primary VUV photons produced in a typical Gerda background event can be enormous. Coincident energy depositions due to ${\beta }$ and ${\gamma }$ interactions in LAr (e.g. from ${}^{228}$Th or ${}^{238}$U trace impurities) frequently reach MeV-energies. The computational effort to track all $\mathcal {O}$($10^{4}$) optical photons represents a challenge, especially when considering the feedback between rejection power and required statistics – the larger the coincident energy release, the larger the suppression, the larger the statistics required to obtain a proper prediction of the HPGe spectrum after veto application. However, there is a workaround for this problem: the light propagation can be separated from the simulations that provide the energy depositions in the LAr.

The number of primary photons $n'$ generated from a single energy deposition $(E,\vec {x})$ in the LAr, follows a Poisson distribution $\mathcal {P}_{n'}(\lambda ) = \lambda ^{n'}e^{-\lambda }/n'!$ with expectation value $\lambda = E \cdot L'$. Each of these photons has the opportunity to get detected with a photon detection probability $\xi (\vec {x})$ , specific for interaction point $\vec {x}$. Accordingly, the number of detected photons *n*, is the result of $n'$ Bernoulli trials, and stays Poisson distributed with expectation value $E \cdot L' \cdot \xi (\vec {x})\,$. Given a full event, with total coincident energy in the LAr distributed over several interaction points $(E_i,\vec {x_i})$, the probability mass function (*pmf*) $\lambda _s[n]$ for the total number of signal photons $n=\sum _i n_i$ reads

1 $$\begin{aligned} \lambda _s [n] = \mathcal {P}_n \Bigg ( \sum _i E_i \cdot L' \cdot \xi (\vec {x_i}) \Bigg ). \end{aligned}$$

As the convolution of several independent Poisson processes, it stays a Poisson distribution described by the sum of the expectation values. Provided that $\xi (\vec {x})$ is known, veto information can be provided on the basis of the underlying energy depositions $(E_i,\vec {x_i})$, and does not require optical simulations. It relies on the assumption that each set of photons, born from a particle’s energy depositions $E_i$, solely depends on the primary light yield $L'$ and is emitted isotropically.^2^

Experiments using scintillation detectors traditionally quote the yield of detected photo-electrons (p.e.) per unit of deposited energy, i.e. the experimental light yield, in e.g. p.e./keV. This number is only meaningful, when considering a homogeneous detector, with uniform response over most of its volume. By construction this is not the case for the Gerda LAr light read-out system, whose purpose is to detect light that emerges from within and around the optically dense HPGe detector array. With this in mind, good veto performance does not necessarily go hand-in-hand with maximum experimental light yield, especially when considering background sources (e.g. residual natural radioactivity of the HPGe detector support structure) that deposits energy in the “darkest” corners of the array (e.g. between an HPGe detector and its holder plate) where the detection probabilities are minimal, or even zero. Hence, it is necessary to determine the full three-dimensional map of light detection probabilities $\xi (\vec {x})$ , with special emphasis on the areas where little light is collected from. This can only be done in a dedicated Monte Carlo study, that takes into account the full photon detection chain of the Gerda LAr instrumentation.

**Fig. 2:**
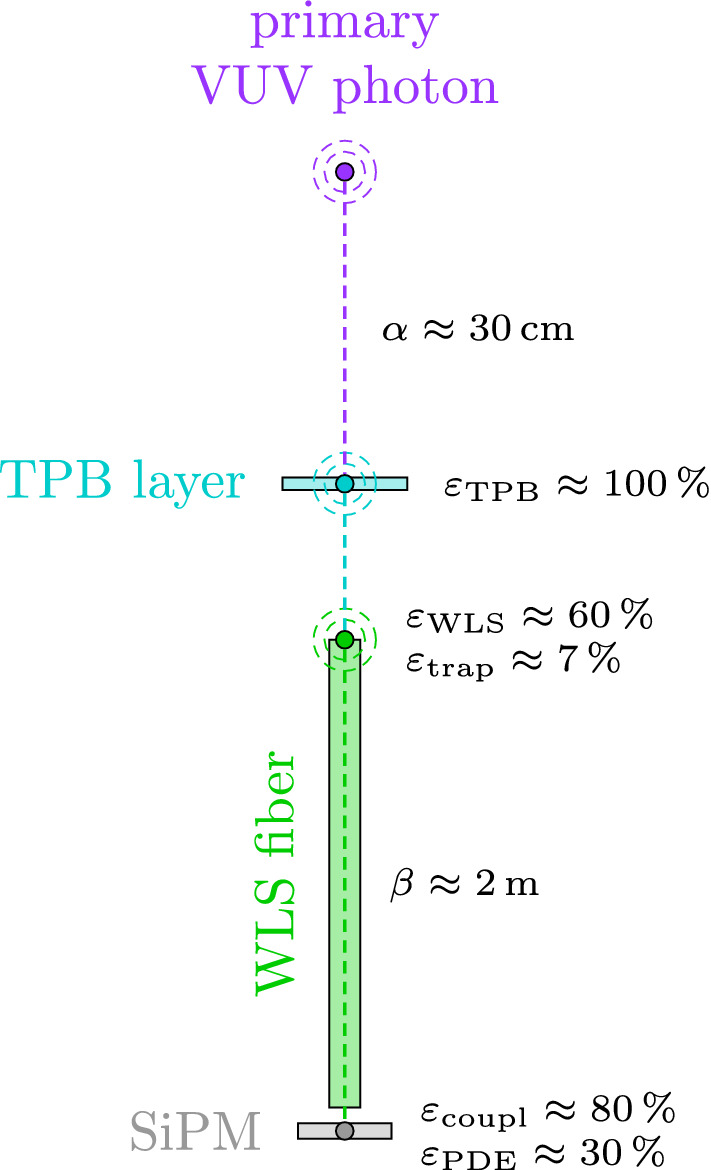
Simplified light collection chain. This one-dimensional representation depicts the main material properties that affect the light collection with the Gerda fiber-SiPM instrumentation. The overall light collection efficiency for the primary VUV photon is of $\mathcal {O}$(0.1)%. In real life, effects like shadowing, reflections and optical coverage enter the game

### A simple estimate

Before running such simulations, it is worthwhile to evaluate the impact of the various steps a primary VUV photon undergoes until its detection. Gerda uses a hybrid system consisting of TPB-coated WLS-fibers with SiPM-readout and PMTs to detect the LAr scintillation light that emerges from in and around the HPGe detector array. If we neglect most geometric effects, the photon detection probability can be broken down into factors that depend on basic properties of the materials and components involved. Given a primary photon that is emitted in the system LAr-TPB-fiber-SiPM, as depicted in Fig. 2, the probability $\xi$ for its detection, can be described by

$$\begin{aligned} \begin{aligned} \xi \;\propto \;&\overbrace{e^{-x/\alpha (\lambda )}}^{\text {LAr}} \;\times \;\varepsilon _{\text {TPB}}(\lambda ) \;\times \; \overbrace{\varepsilon _{\text {WLS}}(\lambda )\;\varepsilon _{\text {trap}}\;e^{-y/\beta (\lambda )}}^{\text {fiber}} \\ \times \;&\underbrace{\varepsilon _{\text {coupl}}\;\varepsilon _{\text {PDE}}(\lambda )}_{\text {SiPM}}, \end{aligned} \end{aligned}$$

where $\lambda$ is the photon wavelength. First, the VUV photon has to travel a certain distance *x* in LAr, while risking to get absorbed in interactions with residual impurities. The absorption length $\alpha (\lambda ={128}\,\textrm{nm})$ at LAr peak emission is on the order of tens of centimeters, depending on the argon purity [16, 18, 19]. The moment the VUV photon reaches and gets absorbed in any TPB layer, a blue photon with peak emission at 420 nm is re-emitted [20]. The efficiency $\varepsilon _\text {TPB}$ for this process is close to 100% [9, 21]. Since the typical distance for a first encounter with a TPB-coated surface is of similar order as the absorption length itself, about 1/*e* of the primary photons make it through this first part of the journey. Once a photon is shifted to blue, absorption in the LAr becomes negligible, as the absorption length for visible light exceeds the actual system size. Hence, it does not necessarily matter, if the blue photon directly enters a fiber at this point or later. As soon as this is the case, the photon undergoes a second wavelength-shifting step and is shifted to green with peak emission at 494 nm [22]. The corresponding efficiency $\varepsilon _\text {WLS}$, i.e. the overlap between the TPB emission and fiber absorption spectrum, is about 60%. The green photon will stay trapped within the fiber with a trapping efficiency $\varepsilon _\text {trap}$ of about 7% [22] and arrive at its end after about half of its absorption length of $\beta \approx {2}\,\textrm{m}$, which adds another factor $1/\sqrt{e}$. The coupling efficiency $\varepsilon _\text {coupl}$ to successfully couple the photon into the SiPM is assumed to be ${80}{\%}$, whereas the photon detection efficiency (PDE) of being detected as a photo-electron signal is about 30% at the green fiber emission [23]. Multiplication of all individual contributions results in an overall detection probability of not more than 0.2% and it can be anticipated that including geometric effects (e.g. shadowing or optical coverage) the light collection will not exceed 0.1% for most regions of the Gerda LAr volume.

## Monte Carlo implementation

The Gerda instrumentation is implemented in the Geant4-based [24–26] Majorana-Gerda (MaGe) simulation framework [27]. For what concerns the propagation of optical photons from typical background processes, most important are the geometries enclosed by the LAr veto instrumentation as well as the optical properties of the corresponding materials.

### Geometry

The HPGe detector array, including all auxiliary components, is implemented to the best available knowledge, but making reasonable approximations. The reader may find detailed technical specifications such as dimensions and materials documented in [7]. The simulated setup includes: individually sized and placed HPGe detectors in their silicon/copper mounts, TPB-covered nylon mini-shrouds around each string, high-voltage and signal flat cables running from each detector to the front-end electronics, the front-end electronics themselves as well as copper structural components. Approximations are made when full degeneracy of events originating from the respective parts is expected, e.g. the level of detail of the electronics boards is low and no detailed cable routing is implemented. Details are discussed in [28]. As a consequence, shadowing effects that impact the optical photon propagation, but not the standard background studies, may not be captured perfectly.

The PMTs are implemented as cylinders, with a quartz entrance window and a photo-sensitive cathode. They are placed at their respective 9(7) positions in the top(bottom) copper plate, which to the inside is covered with a specular reflector that emulates VM2000. In contrast, the inside of the copper shrouds is lined with a diffuse PTFE reflector that represents the  foil. All reflector surfaces, as well as the PMT entrance windows, are covered with a wavelength-shifting TPB layer.

The fiber shroud is modeled as $15\times 6 = 90$ cylinder segments covering the central part of the veto volume. Every segment contains a core, two claddings and one thin TPB layer, just as the real fibers. Their bottom ends have reflective surfaces attached, whereas optical photons reaching the upper ends are registered by photosensitive surfaces, each of them representing one SiPM. This differs from the real-world implementation, where the fibers are bent, up-routed and read-out on both ends. To avoid any misinterpretation of in-fiber correlations between channels that are connected to the same fibers, both the Monte Carlo and data signals are re-grouped to represent one channel per fiber module, resulting in a total of 9 fully independent channels. Due to sagging and uneven tensioning the optical coverage of the fibers was reduced in comparison to the maximum possible value of 75%. In the simulation, a gap between the fiber segments parametrizes the coverage of the fiber shroud. Analyzing photos of the mounted fiber modules the real coverage was estimated to be around 50%.

**Fig. 3:**
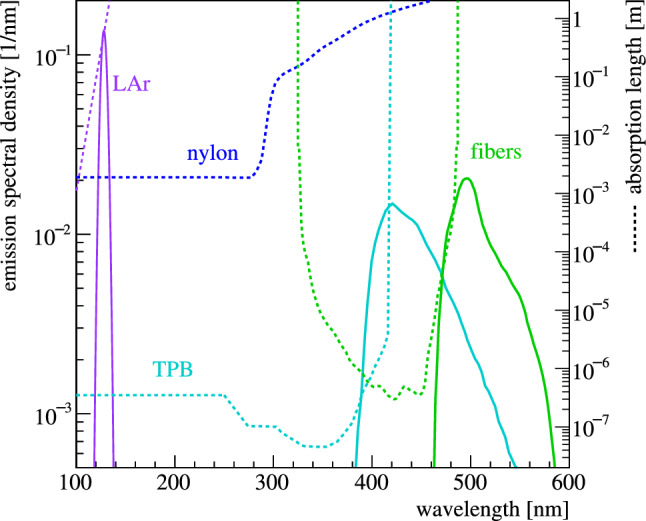
Emission spectra (solid lines) and absorption length (dashed lines) of indicated materials. The primary emission from the LAr follows a simple Gaussian distribution centered at 128 nm. Its absorption length connects to larger wavelength with an *ad-hoc* exponential scaling. Absorption and re-emission appears in TPB and the polystyrene fiber material. Nylon is only transparent to larger wavelength

### Optical properties

Figure 3 compiles the relevant emission and absorption features implemented for the various materials. The emission of VUV scintillation photons from the LAr follows a simple Gaussian distribution centered at 128 nm with a standard deviation of 2.9 nm. It neglects contributions at longer wavelength, which have orders of magnitude lower intensity for pure LAr [29]. The refractive index of the LAr is implemented using the empirical Sellmeier formalism, with the coefficients obtained in [30]. Building on this, the wavelength-dependent Rayleigh scattering length is derived [31]. It corresponds to about 70 cm at LAr peak emission, which is shorter than recently suggested [32]. Operation of the LEGEND Liquid Argon Monitoring Apparatus (LLAMA) during the Gerda decommissioning point towards a VUV attenuation length of about 30 cm [33]. Accordingly, the absorption length was set to $1/(1/30-1/70) \approx {55} \;{\hbox {cm}}$. The absorption length is modeled over the full wavelength range extending it from 128 nm with an *ad-hoc* exponential function. The primary VUV scintillation yield $L'$ is considered a free parameter and by default set to 28 photons/keV. A dependence of the photon yield on the incident particle, i.e. quenching, as well as characteristic singlet and triplet timing are implemented. The TPB absorption length is taken from [9], the emission spectrum from [20]. Individual emission spectra, where available, are implemented for TPB on nylon [13], VM2000 [34] as well as  [10]. The absorption length of nylon is taken from [35]. Absorption and emission of the fiber material use the data presented in [22], normalized to measurements at 400 nm. The quantum efficiency of the PMTs [36] and PDE of the SiPMs [23] have been extracted from the product data sheets provided by the vendors. The reflectivities of germanium, copper, silicon and PTFE above 280 nm are taken from [37], whereas their values at VUV wavelength are largely based on assumptions. The reflectivity of VM2000 is taken from [34], the one of  from [38]. The exact optical property values implemented in the simulation have been reported in [39].

### Uncertainties

A priori , the bare simulations are not expected to reproduce the data. Details like partially inactive SiPM arrays, coating non-uniformities and shadowing by real-life cable management are not captured by the Monte Carlo implementation. Similarly, input parameters measured under conditions differing from those in Gerda, e.g. at room temperature or different wavelength, pose additional uncertainty.

Back to Eq. 1 and photon detection probabilities $\xi (\vec {x})$ : as already the number of primary VUV photons is uncertain, any linear effect, constant across the LAr volume $\vec {x}$, is degenerate with the primary light yield $L'$ and thus only the product $L' \cdot \xi (\vec {x})\,$ can be constrained by data-Monte Carlo comparison. It follows that, if a primary light yield of $L' = {28} \;{\textrm{photons}/\hbox {keV}}$ is assumed, its true value is fully absorbed in a global scaling of the efficiencies $\varepsilon _i$, individually to each light detection channel *i*. The set $\varepsilon _i$ may further absorb any other global effect, e.g. an inaccurate TPB quantum efficiency, as well as any local channel-specific feature, e.g. varying photon detection efficiencies of the photo sensors. Given the large set of potential uncertainties, $\varepsilon _i$ are treated unconstrained and may take any value between zero and unity.

## Parameter optimization

To obtain a predictive model of the performance of the LAr veto system, residual degrees of freedom must be removed. In the following, we describe the methodology employed to statistically infer the value of the efficiencies $\varepsilon _i$ by comparing simulated to experimental data. The full evidence of the model parameters is contained in a likelihood function, which has been maximized for special calibration data.

Given a class of events, the *pmf* $\varLambda [n]$ that describes the number of photons *n* detected by some LAr veto channel is the convolution of two contributions:

2 $$\begin{aligned} \varLambda [n] = \varLambda _s[n] * \varLambda _b[n]\;. \end{aligned}$$

It is a simultaneous measurement of light from true coincidences $\varLambda _s[n]$ that accompany the corresponding HPGe energy deposition as well as random coincidences $\varLambda _b[n]$ largely produced by spectator decays such as e.g. ${}^{39}$Ar in the LAr.^3^ While $\varLambda _s[n]$ may be provided from simulations, randomly triggered events allow an evaluation of $\varLambda _b[n]$ from data. However, as the measured signal amplitudes suffer non-linear effects, e.g. afterpulsing and optical crosstalk, that are themselves under study and at present not implemented in the simulation, no direct *pmf* comparison is possible and instead the binary projection of Eq. 2 is used. In the binary “light/no-light” projection, where $\overline{\varLambda }=\varLambda [0]$ corresponds to no light, and $\varLambda$ to a positive light detection, the *pmf* breaks down to a single expectation value, given by

3 $$\begin{aligned} \begin{aligned} \varLambda&= \varLambda _s \cdot \overline{\varLambda }_b + \overline{\varLambda }_s \cdot \varLambda _b + \varLambda _s \cdot \varLambda _b = \varLambda _s \vee \varLambda _b \\ \overline{\varLambda }&= 1 - \varLambda = \overline{\varLambda }_s \cdot \overline{\varLambda }_b\;. \end{aligned} \end{aligned}$$

A positive light detection is either truly coincident without random contribution, fully random or a simultaneous detection of both. It is complementary to no detection, neither as true nor as random coincidence. $\varLambda _s$($\varLambda _b$) is the detection probability for true(random) coincidences and $\overline{\varLambda }_s = \overline{\varLambda } / \overline{\varLambda }_b$ quantifies the true survival probability of the underlying class of events, corrected for random coincidences.

Even though the data is reduced to binary information, the simulated *pmf* $\varLambda _s$ allows the additional detection efficiency $\varepsilon$ to be folded into the Monte Carlo expectation. Given a count of *n* photons in the bare simulation, an effective detection of $m<n$ photons can be represented as a sequence of Bernoulli trials with probability $\varepsilon$. The *pmf* $\varLambda _s[m](\varepsilon )$ is the result of binomial re-population throughout all $n \ge m$:

4 $$\begin{aligned} \varLambda _s[m](\varepsilon ) = \sum _{n \ge m} \varLambda _s[n] \left( {\begin{array}{c}n\\ m\end{array}}\right) \varepsilon ^m (1-\varepsilon )^{n-m}. \end{aligned}$$

**Fig. 4:**
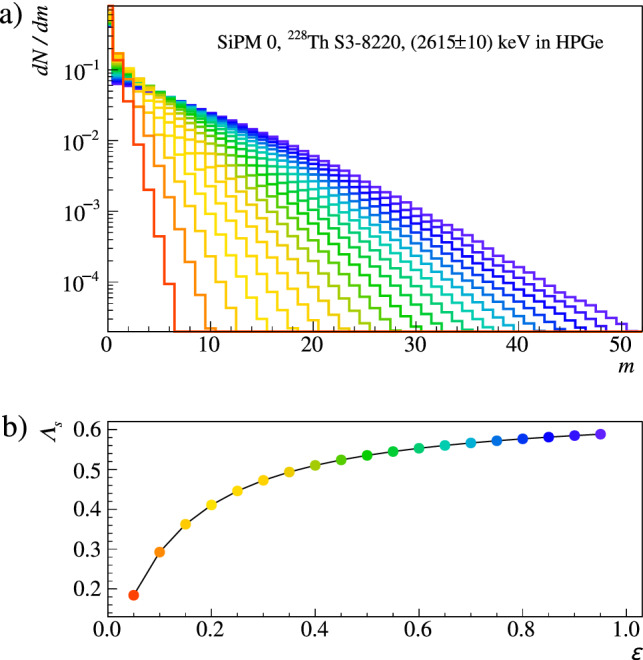
Binomial repopulation. **a** The *pmf* $\varLambda _s[m](\varepsilon )$ defined in Eq. 4 can be obtained for any value of the detection efficiency $\varepsilon$ from the unaltered simulation output. The example shows the *pmf* for a specific SiPM channel as obtained for ${}^{228}$Th decays in a calibration source at the top of the array (see also Tab. 1) depositing 2615 ± 10 keV in the HPGe detectors. The result is plotted for a selection of efficiency values, reported in the bottom panel. **b** The panel shows the non-linear dependence of the light detection probability $\varLambda _s$ (defined in Eq. 5). The color coding relates data points with corresponding distributions in the top panel

This technique avoids re-simulation for different values of $\varepsilon$. Figure 4 depicts Monte Carlo spectra processed for different efficiencies. Back in binary space, the detection probability $\varLambda _s(\varepsilon )$, i.e. the chance to see one photon or more as true coincidence, is

5 $$\begin{aligned} \varLambda _s(\varepsilon ) = 1 - \overline{\varLambda }_s(\varepsilon ) = 1 - \sum _{n} \varLambda _s[n] (1-\varepsilon )^{n} \;. \end{aligned}$$

It is the inverse of no detection, i.e. the population of the “zero bin” $\varLambda _s[0](\varepsilon )$ in Eq. 4 and allows uncertainties on the bare frequencies $\varLambda _s[n]$ to be propagated into $\varDelta \varLambda _s(\varepsilon )$.^4^

The likelihood for the observation of light in *N* Monte Carlo events out of $N_\text {tot}$ simulated in total, given the aforementioned expectation value $\varLambda (\varepsilon )=\varLambda _s(\varepsilon ) \vee \varLambda _b$, is described by a binomial distribution $$\mathcal {B}^N_{N_\text {tot}}(\varLambda ) = \left( {\begin{array}{c}N_\text {tot}\\ N\end{array}}\right) \varLambda ^{N} (1-\varLambda )^{N_\text {tot}-N}$$. Maximizing its value allows to infer on $\varepsilon$, whereas taking into account the limited statistics of the random coincidence dataset, with *M* light detections over $M_\text {tot}$ random events, makes it a combined fit of both the data and the random coincidence sample. This combined likelihood reads:

6 $$\begin{aligned} \begin{aligned} \mathcal {L}(\varepsilon ;\sigma ) = \;&\mathcal {B}_{N_\text {tot}}^{N}\big ((\varLambda _s(\varepsilon )+\sigma \cdot \varDelta \varLambda _s(\varepsilon ))\vee \varLambda _b\big ) \\&\quad \times \mathcal {B}_{M_\text {tot}}^{M}\big (\varLambda _b\big ) \times \hat{\mathcal {G}}\big (\sigma \big ) \;. \end{aligned} \end{aligned}$$

The signal expectation is given flexibility according to its uncertainty $\varDelta \varLambda _s$ using a Gaussian pull term $\hat{\mathcal {G}}(\sigma ) = e^{-\sigma ^2/2}$, which accounts for limited simulation statistics and additional systematics. Equation 6 has zero degrees of freedom and hence model discrimination can only be obtained through a combination of multiple datasets, i.e. calibration source positions, or by exploiting channel event correlations. While the former sounds trivial, e.g. an absorption length can be estimated from measurements at different distance, the latter requires explanation. Let’s imagine two photosensors $i \in \{A,B\}$, each probing the LAr with a certain photon detection probability $\xi _i(\vec {x})$. Considering an energy deposition $(E,\vec {x})$, the probability to see light in both channels depends on $\xi _A(\vec {x}) \cdot \xi _B(\vec {x})$, while events triggering only channel *A* test $\xi _A(\vec {x}) \cdot (1-\xi _B(\vec {x}))$. Both of them probe distinct regions of the LAr volume. The probability to see no light from a certain volume of the LAr, i.e. the corresponding survival probability, depends on $\prod _i (1-\xi _i(\vec {x}))$.

Given the full set of veto channels *S* of size *n*, each event will come as a certain subset, i.e. pattern, $P \subseteq S$ of triggered channels. The total number of possible patterns is $2^n$, where each of them comes with its own unique expectation value derived from signal as well as random coincidences. A pattern’s signal expectation $\varLambda _s(\vec {\varepsilon })$ can be evaluated much like Eq. 4, however starting from an *n*-dimensional hyper-spectrum evaluated for the full vector of efficiencies $\vec {\varepsilon }$. When folding in the random coincidences, it has to be considered that a certain pattern $P_s=\{A,B\}$ may be elevated to e.g. $P=\{A,B,C\}$ by random coincidences of the form $P_b=\{A,C\}$ or similar. Each pattern occurrence expectation value is hence a sum over all possible generator combinations $G=\{P_s,P_b\}$, that result in $P_s \vee P_b = P$. The full likelihood reads

7 $$\begin{aligned} \mathcal {L}(\vec {\varepsilon };\sigma )= & {} \prod _{P} \mathcal {B}_{N_{tot}}^{N}\big (\sum _{G}(\varLambda _s(\vec {\varepsilon })+\sigma \cdot \varDelta \varLambda _s(\vec {\varepsilon }))\cdot \varLambda _b\big ) \nonumber \\{} & {} \times \prod _{G} \mathcal {B}_{M_{tot}}^{M}\big (\varLambda _b\big ) \times \hat{\mathcal {G}}\big (\sigma \big ). \end{aligned}$$

The number of degrees of freedom is $(2^n-n-1)$, where *n* is the number of channels. Several datasets may be combined as the product of the individual likelihoods. Equation 7 implicitly includes correlations between photosensors, which avoids a potential overestimation of the overall veto efficiency that could arise from unaccounted systematics. Given a large set of channels the number of possible patterns may be immense, but can be truncated by e.g. neglecting events with detection pattern higher than a certain multiplicity.

**Table 1:** Calibration data taken with a <2kBq ${}^{228}$Th source placed at different heights. The reported position corresponds to the absolute distance moved from the parking position on top of the experiment. The upper-most and lower-most HPGe detectors are situated at about 8180 and 8560 mm respectively

Position	Live time	Random
[mm]	[h]	Coincidences
8220	6.4	7.5( 6)%
8405	4.3	7.2(10)%
8570	3.6	10.2(14)%

### Application

In order to constrain the aforementioned effective channel efficiencies $\varepsilon _i$, dedicated data taken under conditions that could be clearly reproduced with a Monte Carlo simulation was needed. As the Gerda background data is a composition of various contributions and itself under study, only the peculiar conditions of a calibration run, where the energy depositions originate from a well-characterized source, allow for such studies. However, usual calibrations were taken with the purpose to guarantee a properly defined energy scale of all HPGe detectors and were performed with three ${}^{228}$Th calibration sources of $\mathcal {O}$(10) kBq activity each. The resulting rate in the LAr was far too high to study the veto response and was hence not even recorded during those calibrations. For this reason, special calibration data using one of the former Phase I ${}^{228}$Th sources with an activity of < 2 kBq was taken in July 2016. The source was moved to 3 different vertical positions (see [40] for details about the calibration system). The characteristics of the datasets are compiled in Tab. 1. To avoid ${\beta }$ particles contributing to the coincident light production and enable a clean ${\gamma }$ -only signature, an additional 3 mm copper housing was placed around the source container.^5^ Each configuration was simulated with e8 primary decays in the source volume.

**Fig. 5:**
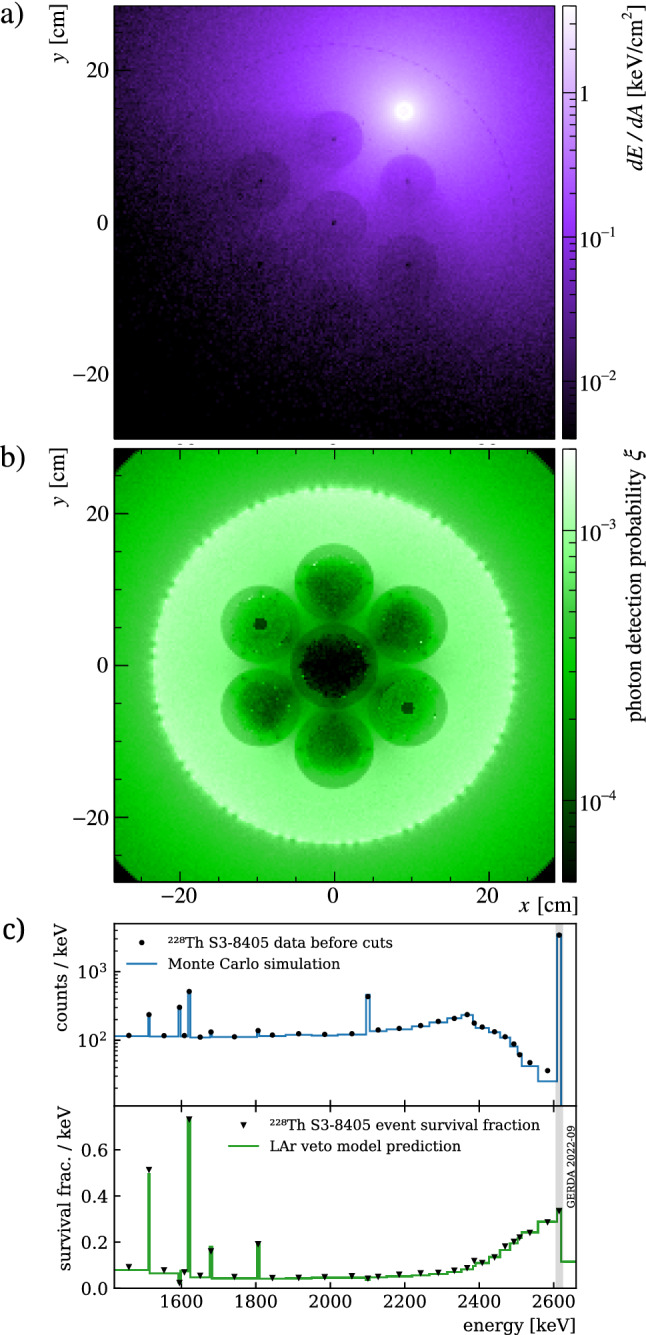
Data/Monte Carlo comparison. **a** Projected distribution of the LAr energy depositions for simulated ${}^{208}$Tl 2615 keV FEP-events from a calibration source at position 8405 mm. Darker circles correspond to the volume occupied by the HPGe detectors. **b** Photon detection probability $\xi$ in the same region. **c** Top panel: the energy spectrum of the ${}^{228}$Th data corresponding to figure **a**, before the LAr veto cut, compared to the Monte Carlo prediction. The *pdf* is normalized to reproduce the total count rate in data. Despite small shape discrepancies, the predicted LAr veto survival probability (bottom panel) matches the data over a wide range of energies, even far from the model optimization energy region (gray band). A variable binning is adopted for visualization purposes

The maximum likelihood analysis was performed on ${}^{208}$Tl full energy peak (FEP) events with an energy deposit of 2615(10) keV in a single HPGe detector. As no direct ${\beta }$ transitions to the ground nor first excited state of the ${}^{208}$Pb daughter nucleus are allowed, a minimum of 3.2 MeV is released in ${\gamma }$ ’s, which almost always includes a transition to the intermediate 2615 keV state. Selecting full absorption events of the corresponding ${\gamma }$ , results in an event sample virtually independent on HPGe detector details – the HPGe array is solely used to tag the ${}^{208}$Tl transition. The coincident energy depositions in the LAr originate to a large extent from coincident ${\gamma }$ ’s of 583 keV or more, and only marginally from *Bremsstrahlung*. Figure 5a shows their energy distribution in the LAr. The random coincidence samples were obtained by test pulse injection at 50 mHz as well as an early ($-20$ $\upmu \hbox {s}$) evaluation of the veto condition. The random coincidence appearance is unique to each source configuration as the energy depositions from independent decays in the source contribute.

To reduce the dimensionality of Eq. 7, the top/bottom PMT channels were regrouped to represent one single large top/bottom PMT, whereas the generally less uniform fiber/SiPM channels were kept separate. Accordingly, the likelihood had to be evaluated in $2 + 9 = 11$ dimensions. The pattern-space was truncated so that only channel combinations present in data had to be calculated. The extracted channel efficiencies are 13% for the top PMTs, 29% for the bottom PMTs and reach from 21 to 37% for the SiPM channels, with uncertainties of about ± 1%. Given an additional systematic uncertainty of 20% on the observation of the various veto patterns (i.e. $\sigma = 0.2$ in Eq. 7), the p-value amounts to 0.2. The differences in the SiPM efficiencies match the expectation for problematic channels with potentially broken chips. The reduced value for the top PMTs was anticipated, as additional shadowing effects from cabling are only present at the top.

## Probability maps

To finally evaluate the three-dimensional photon detection probability $\xi (\vec {x})$ , a dedicated simulation of VUV photons sampled uniformly over the LAr volume around the HPGe array was performed. Positive light detections with any photosensor *i* were determined taking into account the efficiencies $\varepsilon _i$ as obtained in Sect. 5.1. For convenience, $\xi (\vec {x})$ is stored as a discrete map, partitioned into cubic “voxels” of size $3 \times 3 \times 3$ mm$^{3}$. This size matches the characteristic scale of the probability map gradients expected in Gerda. Hence, the detection probability $\xi _k$ associated with voxel *k* corresponds to the ratio between positive light detections and total scintillation photons generated in the voxel volume. Figure 5b shows a projection of this object. As outlined in Sect. 3 it allows to determine the expected number of signal photons and the corresponding event rejection probability, solely based on energy depositions in the LAr. Outside the densely packed array $\mathcal {O}$(0.1)%-level values are reached. Figure 5c compares the energy distribution, before and after the LAr veto cut, of the ${}^{228}$Th source data with the model prediction. Small discrepancies are expected from geometry inaccuracies and the modeling of charge collection at the HPGe surface. However, the event suppression (shown in the bottom panel) is reproduced over a wide range of energies far off the ${}^{208}$Tl FEP-events used for model optimization. As expected, the rejection power is reduced for single-${\gamma }$ FEPs without significant coincidences in the decay scheme, e.g. for ${}^{212}$Bi at 1621 keV, and enhanced for the double escape peak (DEP) at 1593 keV, where two 511 keV light quanta leave the HPGe detectors.

### Distortion studies

**Fig. 6:**
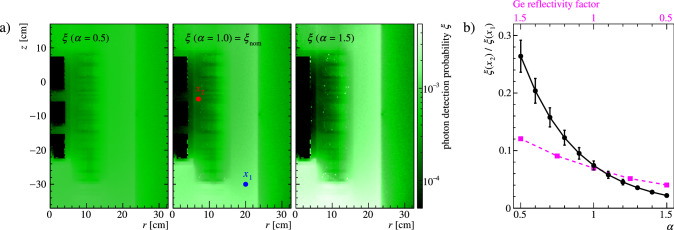
Modification of the photon detection probability $\xi (\vec {x})$ through analytical power-law distortions defined in Eq. 8. **a** Inhomogeneities that are present in the nominal map are amplified with increasing $\alpha$, leading to a more homogeneous ($\alpha <1$, i.e. less color contrast) or less homogeneous ($\alpha >1$, i.e. more color contrast) response. **b** The probability ratio from two sample points $x_1$ and $x_2$, outside and within the array, highlights this modification (black data points). The comparison of an altered germanium reflectivity (magenta data points) shows how the distortions conservatively exceed $\pm \,{50}{\%}$ on the reflectivity

As anticipated in Sect. 4, many uncertainties affect the simulation of the LAr scintillation in the Gerda setup. The channel efficiencies $\vec {\varepsilon }$ extracted from calibration data, as described in Sect. 5, absorb systematic biases that scale the detection probability by global factors (e.g. the LAr scintillation yield and the TPB quantum efficiency), but cannot cure local uncertainties (e.g. due to incorrect germanium reflectivity or fiber shroud coverage). A heuristic approach has been formulated to estimate the impact of such simulation uncertainties on the LAr veto model. The distortion of $\xi (\vec {x})$ induced by varying input parameters can be conservatively parametrized by means of an analytical transformation T:

$$\begin{aligned} \xi (\vec {x})\,\mapsto \xi ^\prime (\vec {x}). \end{aligned}$$

As an additional constraint, the transformed $\xi ^\prime (\vec {x})$ must still reproduce the calibration data presented in Sect. 5, with which the original $\xi (\vec {x})$ was optimized. As a consequence, the LAr volumes probed by calibration data (see Fig. 5) act as a fixed point of the transformation *T*, letting the detection probability deviate from its nominal value in all the other regions of the setup. *T* may take various analytical forms, depending on the desired type of induced local distortions. The adoption of this procedure overcomes the difficulty of studying the dependence of $\xi (\vec {x})$ on numerous optical parameters by performing several computationally expensive simulations of the scintillation light propagation.

In the context of this work, we shall focus on transformations that make $\xi (\vec {x})$ less or more homogeneous, i.e. that make “dark” areas (e.g. the HPGe array) “darker” compared to areas with high detection probability, or *vice versa*. A simple transformation that meets this requirement is the following power-law scaling:

8 $$\begin{aligned} \xi (\vec {x})\,\mapsto N \cdot \xi (\vec {x})\,^\alpha \;, \end{aligned}$$

where $\alpha$ is a real coefficient controlling the magnitude of the distortion and *N* is a normalization constant adjusted to reproduce the event suppression observed in calibration data. The action of the transformation in Eq. 8 on the detection probability is depicted in Fig. 6. In the same figure, the size of power-law distortions is compared to that induced by uncertainties on the HPGe detectors’ reflectivity in the VUV region. The impact of the latter has been evaluated by scaling its value by $\pm \,{50}{\%}$ in dedicated optical simulations. The power-law distortions significantly exceed the effect of a potential reflectivity bias and can therefore be used as a conservative estimate of the uncertainty on the light collection probability.

## Background decomposition

As an application of the light collection and veto model, we shall now present the results of the background decomposition of the HPGe energy spectrum recorded during physics data taking after the application of the LAr veto cut. This background model serves as a fundamental input for various physics analyses whose sensitivity is enhanced with the LAr veto background reduction.

**Fig. 7:**
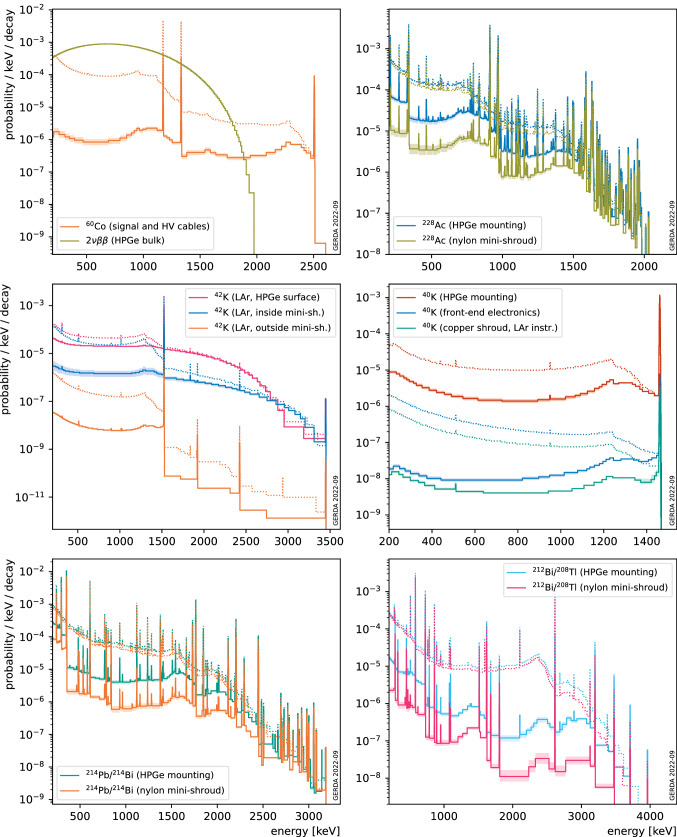
Probability density functions (*pdfs*, normalized to the number of simulated primary decays) for a representative selection of background and signal event sources in the Gerda Phase II setup as detected by HPGe detectors and surviving the LAr veto cut, as predicted by the model presented in this document. Model uncertainties are shown as bands of lighter color. *pdfS* before the cut [28] (dotted lines) are overlaid for comparison. The reader is referred to [28, Figure 1] for a detailed documentation of the simulated setup. A variable binning is adopted for visualization purposes

Previous work [28] has proven successful in describing the Gerda data in terms of background components, but before LAr veto and PSD cuts, referred to as “analysis” cuts. (*pdfs*) for various background sources have been produced with dedicated simulations of radioactive decays in the inner Gerda setup. A linear combination of these *pdfs* has been consequently fit on the first 60.2 kg years data from Gerda Phase II in order to infer on the various contributions to the total background energy spectrum. Since the data was considered before the application of the LAr veto cut, the propagation of scintillation photons has been disabled in these simulations. The developed LAr detector model allows to incorporate this missing information into the existing simulations and compute background expectations after the LAr veto cut. *pdfs* for a representative selection of signal and background sources in the Gerda Phase II setup are reported in Fig. 7.

**Fig. 8:**
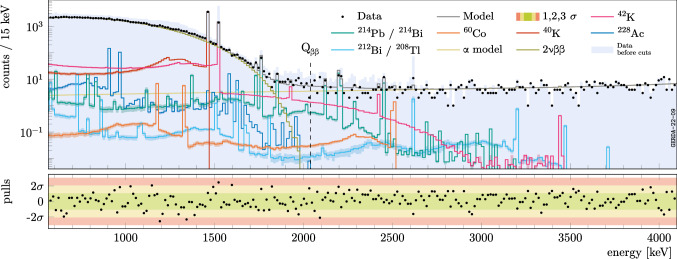
Background decomposition of the first 61.4 kg years of data from Gerda Phase II surviving the LAr veto cut (black dots). The veto model is applied to the existing background *pdfs* before the cut [28] folding in the probability map $\xi (\vec {x})$ . Data before the cut is shown as a light blue filled histogram. Shaded bands constructed with the maximally distorted probability maps provide a visualization of the systematic uncertainty affecting each *pdf*

The LAr veto model is applied to each background contribution before analysis cuts, as established by the background model. The distribution of ${\alpha }$ events originating from the HPGe readout contact is negligibly affected by the LAr veto cut, as the ${\alpha }$ particles are expected to originate from the germanium surfaces themselves, where the light collection is poor and any remaining light output from the recoiling nucleus would be quenched [15]. Thus, the ${\alpha }$ model has been imported as-is from [28]. All predictions after the LAr veto cut are corrected for accidental rejection due to random coincidences of 2.7% [4]. As in [28], a Poisson likelihood is used to compare the detector-type specific energy spectra of single-detector events that survive the LAr veto cut, corresponding to an exposure of 61.4 kg years of Gerda Phase II data,^6^ with a linear combination of background *pdfs*. Statistical inference is carried out to determine the coefficients of the admixture that best describe the data. In a Bayesian setting, posterior probability distributions of background source intensities resulting from the model before cuts are fed as prior information,^7^ with the exception of ${}^{42}$K. The distribution of ${}^{42}$K ions in LAr is knowingly inhomogeneous, as ion drifts are induced by electric fields (generated by high-voltage cables and detectors) and convection. The spatial distribution is at present unknown. As a matter of fact, rough approximations have been adopted to describe it in the background model [28]. Given the inhomogeneity of the LAr veto response itself, a significant mismatch of the predicted event suppression between simulation and data is expected. Hence, we adopt uninformative, uniform priors for the ${}^{42}$K source intensities. The Monte Carlo Markov Chains are run with the BAT software [41] to compute posterior distributions and build knowledge update plots.

Substantial agreement between event suppression predicted by the LAr veto model and data is found: the posterior distributions are compatible with the priors, where non-uniform, at the 1–2 $\sigma$ level. As anticipated, the ${}^{42}$K activity differs significantly from the data before the LAr veto cut. Based on this background decomposition, the event survival probability after LAr veto predicted in the ${0{\nu \beta \beta }}$decay analysis window^8^ is about 0.3% for ${}^{228}$Th, 15% for ${}^{238}$U and 10% for ${}^{60}$Co. As a final remark, we stress that the quoted event suppression in the ${0{\nu \beta \beta }}$decay region can be affected by background modeling uncertainties (e.g. source location, surface-to-volume activity or the exact ${}^{42}$K spatial distribution) whose evaluation is out of the scope of this work. As such, they must be taken *cum grano salis* and can not be generalized for different experimental conditions.

In the energy region dominated by the two neutrino double beta (${2{\nu \beta \beta }}$) decay, i.e. from the ${}^{39}$Ar endpoint at 565 keV to the double beta (${\beta \beta }$) Q-value 2039 keV, excluding the intense but narrow potassium peaks, the ratio between the number of ${2{\nu \beta \beta }}$ events and the residual background influences the sensitivity of searches for ${\beta \beta }$ exotic decay modes [42]. The signal-to-background ratio is about 2 in data before analysis cuts [28] and improves to about 18 after applying the LAr veto cut.

The obtained background decomposition is displayed in Fig. 8. Bands constructed by using maximally distorted maps (i.e. distortion parameter $\alpha = 0.5$ and 1.5), obtained with the procedure described in Sect. 6.1, are displayed for every background contribution to represent the systematic uncertainty. The ratio between data and best-fit model normalized by the expected statistical fluctuation in each bin, is shown below in the bottom panel. No significant deviations are observed, beyond the expected statistical fluctuations.

## Conclusions

This paper describes the methodology, optimization and application of the light collection model as developed for the Gerda LAr scintillation light read-out. It is based on an *ansatz* that decouples the light propagation from non-optical simulations, using photon detection probability maps. The model has been optimized using low-activity ${}^{228}$Th calibration data. It allows predictions of the LAr veto event rejection, which is central for analyses of the ${2{\nu \beta \beta }}$ spectrum [42], including a precise determination of the ${}^{76}$Ge half-life as well as a search for new physics phenomena. Even though detailed insight into the heterogeneous setup was granted, one short-coming seems imminent: certain parts of the probability maps are extrapolated, as they remain largely unprobed by the available calibration data. The systematic uncertainty associated with this problem has been evaluated using analytical distortions of the veto response. It is left to the upcoming Large Enriched Germanium Experiment for Neutrinoless double beta Decay (LEGEND) experiment to reduce this uncertainty by dedicated calibration measurements, that will elevate their LAr instrumentation from a binary light/no-light veto to a full-fledged detector.

## Data Availability

This manuscript has associated data in a data repository. [Authors’ comment: The data shown in Fig. 8 is available in ASCII format as Supplemental Material [43].]

## Footnotes

1 This estimate only considers contaminant-induced non-radiative de-excitation to compete with the triplet decay and neglects impurities that effect the initial excimer production.

2 These assumptions break down in the case of particle-type dependent quenching or Cherenkov radiation emission. Both effects, as well as an explicit Fano factor, could be added to Eq. 1 for future studies. Recent studies suggest the absence of further energy-dependent quenching effects in LAr [17].

3 It is not the pure Poisson distribution of Eq. 1, as it arises from all different realizations of coincident energy deposition in the LAr as $\varLambda [n] = \langle \lambda [n] \rangle$.

4 Defined as $[\varDelta \varLambda _s(\varepsilon )]^2 = \sum _n \{ [(1-\varepsilon )^n - \overline{\varLambda }_s(\varepsilon )] {\varDelta N_n} / N_\text {tot} \}^2$, where $\varDelta N_n$ is the uncertainty of the unaltered *n*-photon observations in a total of $N_\text {tot}$ Monte Carlo events.

5 The suppression achieved in this configuration is not representative for backgrounds detected during physics data taking, which mostly originate from thin low-mass structural components, where ${\beta }$ particles contribute to the energy depositions in the LAr.

6 Additional 1.2 kg years of data from the last physics run of Phase II , neglected in previous works [28], is considered here.

7 The procedure is not statistically rigorous, since data before and after the cut are not fully independent. Nevertheless, the methodology is considered acceptable, as the intention is to carry out a qualitative comparison.

8 The ${0{\nu \beta \beta }}$decay analysis window is defined as the energy window from 1930 keV to 2190 keV, excluding the region around $Q_{\beta \beta }$ ($Q_{\beta \beta }$ $\pm 5$ keV) and the intervals 2104 ± 5 keV and $2119 \pm 5$ keV, which correspond to known ${\gamma }$ lines from ${}^{208}$Tl and ${}^{214}$Bi.
